# Proteomic analysis of *Aspergillus niger* 3.316 under heat stress

**DOI:** 10.1002/mbo3.1012

**Published:** 2020-02-27

**Authors:** Xiangyu Deng, Bin Du, Fengmei Zhu, Yanan Gao, Jun Li

**Affiliations:** ^1^ Hebei Normal University of Science and Technology College of Food Science and Technology Qinhuangdao China

**Keywords:** *Aspergillus niger*, bioinformatics analysis, heat stress, iTRAQ, parallel reaction monitoring, proteomics

## Abstract

β‐Glucosidase production by *Aspergillus niger *is accompanied by an inevitable temperature increase in the industrial fermentation environment. Hence, the synthetic process of β‐glucosidase is negatively affected. However, our understanding of the heat stress response (HSR) mechanism in *A. niger* is still incomplete. The current study explored the intracellular proteome profile of *A. niger* 3.316 in group T (50°C stress) and group C (30°C control) using two proteomic approaches (isobaric tags for relative and absolute quantitation [iTRAQ] and label‐free) and examined the expression of four proteins using a parallel reaction monitoring (PRM) approach. Based on the result of the iTRAQ proteomic analysis, 1,025 proteins were differentially expressed in group T compared to group C. Using the label‐free approach, we only focused on 77 proteins with significant changes in their protein expression levels. In addition, we performed bioinformatics analysis on all these proteins and obtained detailed gene ontology (GO) enrichment and Kyoto encyclopedia of genes and genomes (KEGG) pathway results. Under heat stress conditions, the relative expression levels of proteins with protection and repair functions were upregulated in *A. niger *3.316. These proteins were involved in metabolic pathways, oxidative phosphorylation, porphyrin and chlorophyll metabolism, pyruvate metabolism, and the citrate cycle (TCA cycle). The insights obtained from the presented proteomics and bioinformatics analyses can be used to further explore the HSR mechanism of *A. niger*.

## Graphical abstract

We explored the intracellular proteome profile of *Aspergillus niger* 3.316 in group T (50°C stress) and group C (30°C control) using isobaric tags for relative and absolute quantitation proteomic approach, and the observations were further verified using a parallel reaction monitoring approach. The label‐free proteomic analysis provided an additional explanation of the differences between groups T and C. Bioinformatic analysis, combined with the proteomics data, significantly enhances our understanding of the heat stress response mechanism of *A. niger*.

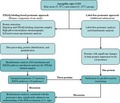

## 1 INTRODUCTION

Currently, *Aspergillus niger* is one of the most important industrial fermentation microorganisms and a significant producer of lignocellulosic enzyme biomass worldwide (Andersen et al., 2011; Souza et al., 2013). Cellulase is used in the degradation of lignocellulosic biomass into soluble free sugars in order to produce ethanol (Adav, Li, Manavalan, & Punt, 2010). It has the potential to generate an alternative clean energy source for various industries (Adav et al., 2010). β‐Glucosidase produced by *A. niger* is an important component of the cellulase enzyme complex and has been widely used in industrial production (Lima et al., 2013).

Liquid media for *A. niger* fermentation are based on an unsteady‐state operation. The fermentation temperature and the flow rate are two significant factors that influence the production of β‐glucosidase (Abrashev et al., 2014). Generally, the optimum temperature for the growth of most fungi is 37**°**C (Klinkert & Narberhaus, 2009; Shankar, Nigam, Saxena, & Madan, 2004). An increase in the temperature can have several consequences for fungal cells, primarily a decrease in cell viability (Bhabhra & Askew, 2005; Lamoth, Juvvadi, Fortwendel, & Steinbach, 2012). *A. niger* is certainly no exception. During the fermentation of *A. niger*, substantial cellular damage can be caused by a temperature increase in the fermentation environment (Abrashev et al., 2008). Consequently, the efficiency of producing β‐glucosidase during industrial fermentation is greatly reduced. To circumvent this disadvantage, the production cost can increase substantially to maintain the relative stability of the fermentation temperature. However, fungal cells have adaptation mechanisms to reduce the impact of heat stress. One of the most significant adaptation mechanisms for fungal cells under heat stress is the heat stress response (HSR), a highly conserved gene expression regulatory circuit that inhibits protein biosynthesis and induces the expression of a series of cytoprotective genes encoding heat shock proteins (HSPs; Verghese, Abrams, Wang, & Morano, 2012). HSPs are soluble intracellular proteins that can increase the protection of fungi from environmentally induced cellular damage, which in turn regulates HSR (Liu, Yang, & Ma, 2007). HSPs are involved in various routine biological processes in fungi, including transcription, translation, and posttranslational modifications (Tiwari, Thakur, & Shankar, 2015). HSPs serve as molecular chaperones to promote refolding of unfolded or misfolded proteins and can help identify proteins that have been denatured (Thomas, Campos, Le, & Guyotat, 2005). Heat stress can also cause the production of reactive oxygen species (ROS; Abrashev et al., 2014). Various ROS‐scavenging proteins can respond to heat stress, such as catalase (CAT), superoxide dismutase (SOD), thioredoxin reductases (TrxRs), and glutaredoxin–glutathione reductases (GRX‐GRs; Zhang, St. Leger, & Fang, 2017). Several protein families, in addition to HSPs, are involved in the process of HSR.

To the best of our knowledge, this is the first study to perform proteomic (isobaric tags for relative and absolute quantitation [iTRAQ] and label‐free) and bioinformatic analysis on the intracellular proteins of *A. niger *3.316 under heat stress. The relative quantification results for the protein samples were estimated in the heat stress (T, 50°C) and control (C, 30°C) groups using the iTRAQ labeling‐based proteomic approach. Because iTRAQ has the advantages of sensitivity and reliability, we regarded the iTRAQ proteomic analysis as the primary component of our study; the label‐free proteomic analysis serves as additional information. Based on the results of the label‐free proteomic analysis, we only focused on 77 proteins with significant changes in their protein expression levels. Bioinformatic analysis was conducted for all differentially expressed proteins (DEPs). Furthermore, parallel reaction monitoring (PRM) is a targeted hypothesis‐driven proteomics approach, offering high sensitivity, reproducibility, and precision (Bourmaud, Gallien, & Domon, 2016). PRM ensures quantitative sensitivity and specificity, with high‐throughput detection, and a strong anti‐interference ability and high resolution in complex backgrounds (Tang et al., 2014; Taumer et al., 2018). To further substantiate our observations from the iTRAQ and label‐free analysis, we examined the expression of selected proteins using a PRM approach.

At present, a limited number of studies have been conducted on *A. niger* HSR. The main studies that examined HSPs and ROS‐scavenging proteins are those by Sorensen, Lametsch, Andersen, and Nielsen (2009) and Abrashev et al. (2008). The number of previously studied proteins associated with HSR is relatively insufficient. A comprehensive proteomic analysis of *A. niger*, in combination with sufficient bioinformatic data, has not been previously reported. The exact process of HSR in *A. niger* remains unknown. Therefore, the aim of the present study was to combine the results of the proteomic and bioinformatic analyses to better understand the mechanism of HSR of the *A. niger *3.316 strain.

## 2 MATERIALS AND METHODS

### 2.1 Chemicals

The chemicals and reagents used in this study included radioimmunoprecipitation assay (RIPA) lysis buffer (89900; Thermo Fisher Scientific), dithiothreitol (DTT) (161‐0611; Bio‐Rad), iodoacetamide (163‐2109; Bio‐Rad), dissolution buffer (4381664; AB Sciex), trypsin (V5111; Promega), iTRAQ 8‐plex (4390812; AB Sciex), acetonitrile (100030; Merck KGaA), and formic acid (56302; Sigma‐Aldrich). In addition, sucrose (V900116), NaNO_3_ (229938), MgSO_4_·7H_2_O (1058860), KCl (746436), FeSO_4_·7H_2_O (F8263), and K_2_HPO_4_ (P8709) were obtained from Sigma‐Aldrich.

### 2.2 Microorganism culture and heat stress conditions

The experimental fungal strain used in the present study, *A. niger* 3.316, originated from the Chinese General Microbiological Culture Collection Center (CGMCC). A preculture was prepared by inoculating *A. niger* 3.316 in 250‐ml conical flasks containing 100 ml of Czapek Dox liquid medium. The medium composition was as follows: 3 g sucrose, 0.3 g NaNO_3_, 0.05 g MgSO_4_·7H_2_O, 0.05 g KCl, 0.001 g FeSO_4_·7H_2_O, and 0.1 g K_2_HPO_4_. These reagents were mixed in 100 ml of distilled water and incubated at 30**°**C at 155 rpm for 60 hr.

The preculture was added (10 ml) to a 250‐ml conical flask containing the Czapek Dox culture medium (100 ml). It has been shown that *Aspergillus niger *26 can survive under heat stress at 45**°**C (Abrashev et al., 2014). Our previous work indicated that 50**°**C is suitable for heat stress, and this temperature was therefore used in the present study. After inoculation, the samples were divided into two groups, the control (C) and treatment (T) groups, cultured at 30**°**C and 50**°**C (stress), respectively, at 155 rpm for 24 hr.

### 2.3 Preparation of protein samples to be analyzed

#### 2.3.1 Protein extraction

The *A. niger* 3.316 samples were divided into two parts, one part for iTRAQ proteomic analysis (four biological replicates) and the other part for label‐free proteomic analysis (three biological replicates). After the heat stress, extraction of the intracellular proteins of *A. niger *3.316 was achieved using RIPA lysis buffer. The samples were then incubated on ice for 30 min with occasional shaking. The insoluble components were removed via centrifugation at 15,000 *g* for 1 hr at 10°C. The protein concentration was measured using the Bradford assay (Harlow & Lane, 2006).

#### 2.3.2 Digestion and iTRAQ labeling of protein samples

Two hundred micrograms of protein in each sample were mixed with 25 mM DTT and incubated at 60℃ for 1 hr (Nel, Garnett, Blackburn, & Soares, 2015). Subsequently, 50 mM iodoacetamide was added (Nel et al., 2015). After 10 min, the mixture was centrifuged for 20 min at 12,000 g (Nel et al., 2015). One hundred microliters of dissolution buffer was added, and the solution was then centrifuged at 12,000 *g* for 20 min; this step was repeated three times. Next, four micrograms of trypsin were added to each protein sample with a ratio of 1/50 (trypsin/protein). All protein samples were digested at 37℃ for 12 hr. The digestion of the proteins was conducted with four replicates. Thereafter, the digested samples were labeled with the iTRAQ 8‐plex according to the manufacturer's protocol (Karp et al., 2010). For the group C samples, the details are as follows: sample C1 (113 tag), C2 (114 tag), C3 (115 tag), and C4 (116 tag). For the group T samples, the details are as follows: sample T1 (117 tag), T2 (118 tag), T3 (119 tag), and T4 (121 tag). Thereafter, the eight labeled samples were combined. The mixed sample was used for chromatography.

### 2.4 High‐pH reversed‐phase chromatography

A RIGOL L‐3000 HPLC system (RIGOL, China) with a Durashell‐C18 column (4.6 mm × 250 mm, 5 μm, 100 Å) (Agela, USA) was used to fractionate the iTRAQ‐labeled peptides. One hundred microliters of sample was loaded onto the column in A buffer (98% doubly distilled water, 2% acetonitrile, pH 10) and eluted in a combination of A buffer and B buffer (2% doubly distilled water, 98% acetonitrile, pH 10) with a gradient of 5% to 95% over 73 min, at a flow rate of 0.7 ml/min. A total of 10 fractions were obtained, and mass spectrometry detection was performed on each fraction.

### 2.5 Q Exactive mass spectrometry analysis

Each sample was split into two volumes and subsequently analyzed on an EASY‐Spray C18 LC column (75 μm × 120 mm, 3 μm) (Thermo Fisher Scientific). A Q Exactive mass spectrometer (Thermo Fisher Scientific) combined with an EASY‐nLC 1000 system (Nano HPLC, Thermo Fisher Scientific) was used to analyze the samples. Ten microliters of each sample was separated using mobile phase A (99.9% ultrapure water, 0.1% formic acid) and mobile phase B (99.9% acetonitrile, 0.1% formic acid), with a gradient of 5% to 95% mobile phase B for 126 min, at a flow rate of 0.3 μl/min. Then, the eluate was analyzed using the Q Exactive mass spectrometer.

The MS data were acquired in high sensitivity mode, using the following analytical parameters: ion source EASY‐Spray, spray voltage 2.3 kV; capillary temperature, 320℃; full scan automatic gain control (AGC), target 3E6; resolution, 70,000 (full width at half maximum, fwhm); full scan maximum injection time, 20 ms; and scan range, 300 − 1,800 *m/z*. The MS/MS spectrum parameters were as follows: resolution, 17,500 (fwhm); AGC target, 1E5; maximum injection time, 120 ms; and intensity threshold, 8.30E3. A total of 30 data‐dependent MS/MS scans were acquired for each full scan. Precursor ions were further fragmented in a collision cell using normalized collision energy of 32%, and the fragments were quantitated using an Orbitrap.

### 2.6 Data processing, protein identification, and quantification

All MS and MS/MS data were analyzed by the ProteoWizard software (version 3.0.8789). The MS/MS raw data were searched against the *A. niger* subset of the UniProt database (uniprot‐proteome‐*Aspergillus_niger*‐5061; http://www.uniprot.org/) using the Mascot Distiller software (version 2.6.0; Adav et al., 2010). Each fraction corresponded to one raw file (10 raw files). The mass spectrometry raw data have been deposited to the ProteomeXchange Consortium via the iProX partner repository. Trypsin was selected for protein cleavage site identification, with a maximum of two allowed missed cleavages. Carbamidomethylation at cysteine and iTRAQ 8‐plex labels were set as fixed modifications. Mascot searches were performed using a fragment ion mass tolerance of 0.02 Da and a parent ion tolerance of 10.0 ppm.

The Scaffold software (version 4.6.2) was used for protein identification and quantification. Moreover, Scaffold was used to further filter the database search results with a 1% false discovery rate (FDR) at the protein level and two unique peptides per protein (Searle, 2010). Following data filtering, the peptide abundance of the different reporter ion channels of the MS/MS scan was normalized. The protein abundance ratio was based on unique peptide results. Furthermore, the relative quantitation results (ratio) of all identified proteins were obtained according to channels 117/113, 118/114, 119/115, and 121/116, corresponding to T1/C1, T2/C2, T3/C3, and T4/C4, respectively. Proteins with a fold change greater than or equal to 1.3 (T/C ≥ 1.3) or T/C ≤ 0.83, with a *p*‐value < .05 by Student's *t* test, were selected as DEPs. A volcano plot was constructed by combining the fold‐change analysis and *t* test results using the Perseus software (version 1.5.5.1; Tyanova, Temu, Sinitcyn, et al., 2016).

### 2.7 Bioinformatic analysis for iTRAQ‐labeled samples

Hierarchical clustering analysis was carried out using the Perseus software (version 1.5.5.1) to identify proteins with a significant fold change between groups T and C (Tyanova, Temu, Sinitcyn, et al., 2016). GO enrichment was performed using the Blast2GO software (version 4.1; Conesa et al., 2005). All DEPs were subjected to KEGG pathway enrichment analysis using the Koba software (version 2.0, a database for KEGG; Xie et al., 2011). KEGG pathways with computed *p*‐values <.05 were considered significantly enriched. In addition, the proteins selected based on their expression profile under heat stress conditions were also screened using GO enrichment analysis (Blast2GO software, version 4.1).

### 2.8 Label‐free proteomic analysis and bioinformatic analysis

Label‐free proteomic analysis was also performed on the samples of groups T and C. The analysis included three biological replicates for each group (6 samples). The experimental procedures for the label‐free proteomic analysis of the samples were identical to those described for the iTRAQ proteomic analysis, except for the iTRAQ labeling and chromatography steps, which were not executed. MS detection was performed on each sample, where each sample generated one raw file. The mass spectrometry raw data have been deposited to the ProteomeXchange Consortium via the iProX partner repository. Moreover, MaxQuant (version 1.6.2) was used for protein identification according to the following settings (Cox & Mann, 2008; Tyanova, Temu, & Cox, 2016): trypsin cleavage with a maximum of two missed cleavages; peptide mass matching error tolerance of 10 ppm; fragment mass tolerance of 0.05 Da; fixed carbamidomethylation of cysteine; variable modifications of deamidation of asparagine, deamidation of glutamine, and oxidation of methionine. GO enrichment analysis was also performed using the Blast2GO software (version 4.1).

### 2.9 Parallel reaction monitoring and data processing

PRM was performed to further verify the MS observations. The *A. niger* 3.316 samples used for PRM were the same as those used for proteomic analysis (iTRAQ and label‐free). The biological replicates used for PRM were also the same as those used to perform the discovery‐phase experiment. We selected three of the 53 upregulated proteins (based on iTRAQ analysis, Table 2) for PRM verification: alpha, alpha‐trehalose glucohydrolase TreA/Ath1, GPI‐anchored cell wall organization protein Ecm33, and 60S acidic ribosomal protein P1. We also selected sugar transporter for PRM verification, from the list of 18 proteins studied by label‐free analysis (Table 3). A target peptide list consisting of nine peptides was selected from the four selected proteins, based on a data‐dependent acquisition (DDA) experiment. This target peptide list was then imported into the inclusion list of the Xcalibur PRM module, and a candidate ion mass selection error tolerance of 5 ppm was set for PRM mode detection.

A Q Exactive HF quadrupole Orbitrap mass spectrometer (Thermo Fisher Scientific) combined with a NanoAcuity UPLC (Waters) was used to analyze the samples. The samples were loaded in mobile phase A (99.9% ultrapure water, 0.1% formic acid) and eluted by a combination of mobile phase A and mobile phase B (99.9% acetonitrile, 0.1% formic acid), with a gradient of 2%‒80% mobile phase B for 120 min at a flow rate of 0.3 μl/min. The MS/MS data were acquired using the following parameters: spray voltage, 2.0 kV; capillary temperature, 300°C; resolution, 15,000 (fwhm); maximum ion injection time, 100 ms; and automatic gain control (AGC) target, 1E5. In addition, the parameters were set to account for peptides with two charges and no peptides containing methionine. The candidate parent ion was fragmented using high‐energy C‐trap dissociation (HCD) and scanned using an Orbitrap. The scan range (110–2,000 *m*/*z*) is automatically controlled according to the *m*/*z* ratio of the parent ion. The MS/MS raw data were processed using the Skyline (version 19.1.0.193) software to evaluate the peak shapes of the target peptides. The data were deemed reliable when the peak shape was intact and the retention time was within the set retention time range (Maclean et al., 2010). The peptide abundance of the selected four proteins was then determined. Peptides with computed *p*‐values lower than .1 (two‐tailed Student's *t* test) were considered reliable. The PRM analysis included four biological replicates.

### 2.10 Statistical analysis

Student's *t* test was used to compare the protein quantification data of samples in group C (30°C control) and group T (50°C stress). Fisher's exact test was performed on the data from the GO and KEGG pathway enrichment analyses. The PRM data were analyzed using a two‐tailed Student's *t* test.

## 3 RESULTS

### 3.1 Quantification of intracellular proteins based on iTRAQ labeling

The intracellular expression levels of the proteins of *A. niger *3.316 were markedly affected by heat stress. A total of four independent iTRAQ labeling experiments were performed, and the results demonstrated that the experiments were reproducible. The search results from the Uniprot database indicated that 1,025 proteins were differentially expressed in the samples of group T compared to the samples of group C. Correlation analysis of the protein intensity demonstrated the reliability of the experimental data.

**Figure 1:**
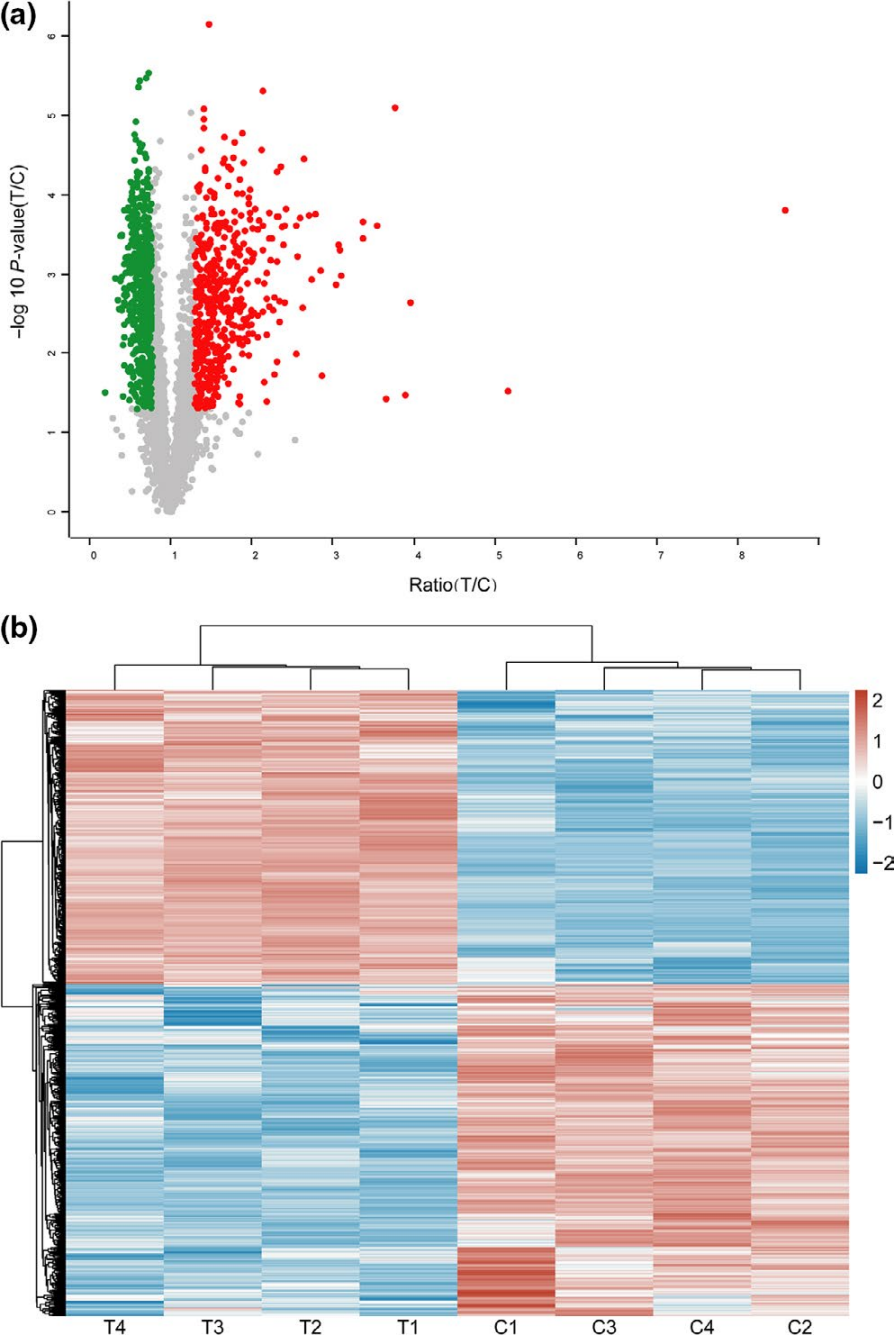
Volcano plot and hierarchical cluster analysis of the 1,025 differentially expressed proteins (DEPs). (a) Volcano plot representation of the identified protein results indicating the ratio (*x*‐axis) and significance (−log10_*p* values, *y*‐axis). The plot indicated that 1,025 proteins were significantly (*p* < .05) upregulated or downregulated (ratio ≥ 1.3 or ≤0.83, respectively) in the T group (50°C heat stress) compared with those of the C group (30°C control). (b) Hierarchical cluster analysis of DEPs in the T group and C group samples. The clustering results are shown for four independent iTRAQ experiments. The C and T groups consisted of four test samples in each group. Downregulation of protein expression is shown in blue; upregulation of protein expression is shown in red

The volcano plot (Figure 1a) simultaneously presents the fold change and *t* test results. The comparison of the quantitative data of the proteins in group C indicated that 481 proteins were highly upregulated in *A. niger *3.316, whereas 544 proteins were downregulated. Hierarchical clustering analysis illustrated that 1,025 DEPs in the T group were clearly distinguished from the proteins in the C group, whereas the ratio of all DEPs in each sample was intuitively represented (Figure 1b). The upregulated proteins were involved in the HSR mechanism of *A. niger *3.316.

### 3.2 Bioinformatic analysis data of the 1,025 DEPs

**Figure 2:**
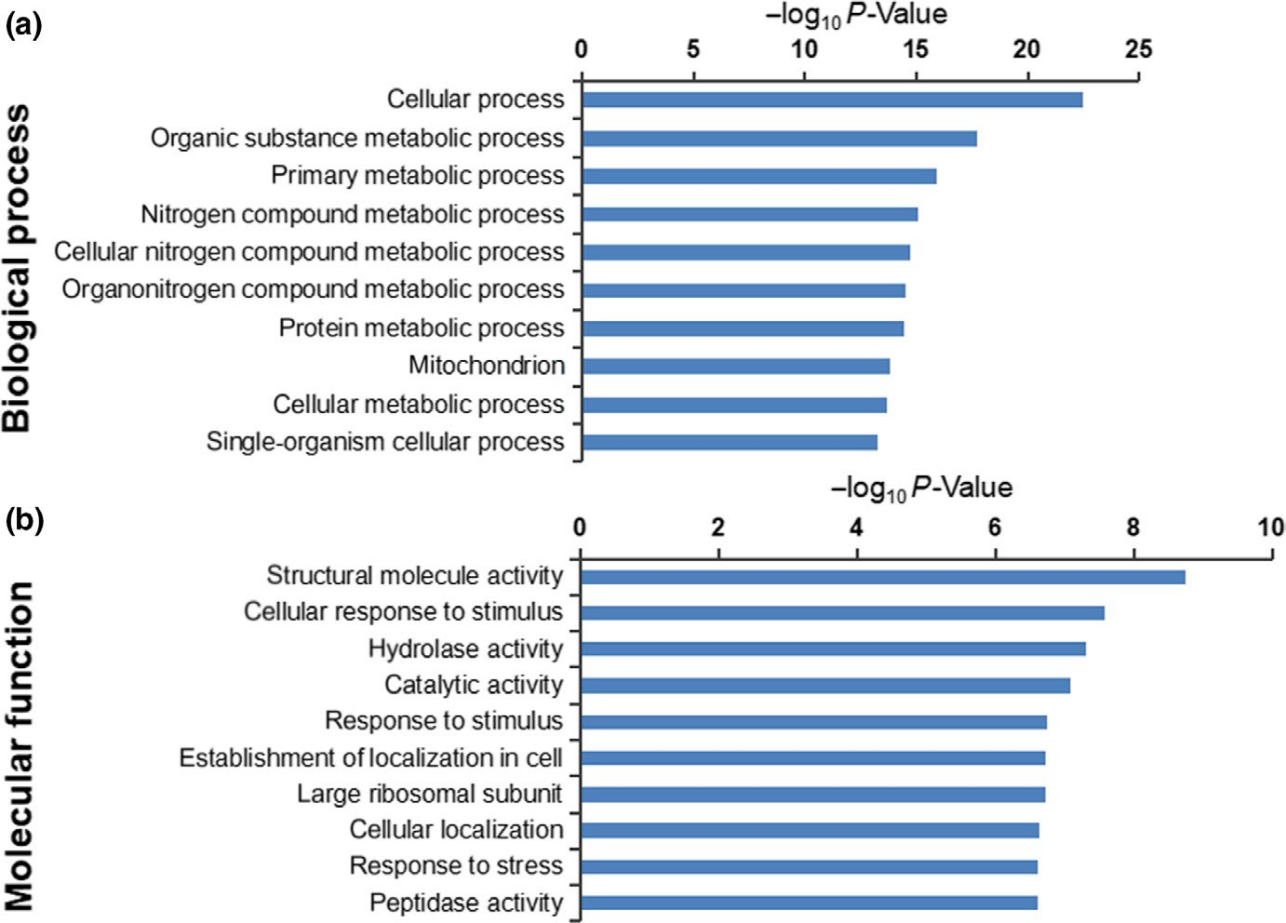
GO enrichment analysis of differentially expressed proteins (DEPs) between the T (50°C heat stress) and C (30°C control) groups. GO enrichment terms (*y*‐axis) and significance (−log10_*p* values, *x*‐axis). (a) Top 10 biological process enrichment terms for the 1,025 proteins. (b) Top 10 molecular function enrichment terms for the 1,025 DEPs

The Uniprot and KEGG database searches allowed us to correlate the GO results with molecular functions and biological processes related to the *A. niger *3.316 HSR. The top 10 most significant terms of protein enrichment were identified by GO molecular function analysis and biological process analysis (Figure 2a,b, respectively).

Importantly, the analysis indicated that certain terms were more closely related to HSR. We further defined eight molecular function terms and five biological process terms (Table 1), including 53 upregulated proteins (Table 2) and 41 downregulated proteins (Table A1). These terms were supported by bioinformatic information metadata on the GO database as follows: The molecular functional (MF) section included cellular response to stress, intracellular protein transmembrane transport, cellular response to misfolded proteins, unfolded protein binding, positive regulation of molecular function, cellular response to topologically incorrect protein, antioxidant activity, and peroxidase activity (Table 1). The biological process (BP) analysis section included protein folding, cellular response to oxidative stress, cellular protein modification process, regulation of macromolecular metabolic processes, and cellular response to heat (Table 1). Certain upregulated or downregulated proteins were involved in multiple GO terms. This resulted in a total selection of 94 proteins (53 upregulated and 41 downregulated proteins), of which 54 were specialized in the cellular response to stress, eight carried out intracellular protein transmembrane transport, four were involved in cellular response to misfolded proteins, eight operated in unfolded protein binding, nine operated in positive regulation of molecular function, and others played key roles in the cellular response to topologically incorrect proteins (five proteins), antioxidantactivity (seven proteins), and peroxidase activity (six proteins). Furthermore, a total of 14, 38, and 14 proteins were involved in protein folding, cellular protein modification process, and cellular response to oxidative stress, respectively, whereas 44 participated in the regulation of macromolecular metabolic processes and nine were specialized in the cellular response to heat. Based on the GO terms presented in Table 1, and the specific ratio of each DEP listed in Table 2, it can be inferred that these proteins may play important roles in the HSR mechanism of *A. niger *3.316.

**Table 1:** GO terms most relevant to the HSR mechanism of *A. niger *3.316

Term	Number of DEPs	*p*‐Value
Molecular function		
Cellular response to stress	54	1.15E−06
Intracellular protein transmembrane transport	8	.015
Cellular response to misfolded protein	4	.016
Unfolded protein binding	8	.021
Positive regulation of molecular function	9	.022
Cellular response to topologically incorrect protein	5	.026
Antioxidant activity	7	.029
Peroxidase activity	6	.031
Biological process		
Protein folding	14	.001
Cellular response to oxidative stress	14	.001
Cellular protein modification process	38	.013
Regulation of macromolecule metabolic process	44	.022
Cellular response to heat	9	.003

**Table 2:** Identification of upregulated proteins in the eight molecular function annotations and five biological process enrichment terms of GO

Protein name	Accession no.	*p*‐Value	Ratio
Alpha, alpha‐trehalose glucohydrolase TreA/Ath1	A0A100I1U6	.00015	2.419
Aha1 domain family	A0A117DZ55	.0025	1.534
Phosphatidyl synthase	A0A117DWL1	.0065	1.338
Kexin	A0A117DWS3	.0014	1.594
UDP‐glucose:glycoprotein glucosyltransferase	A0A100IE92	.0059	1.465
Protein transport protein SEC13	A0A117DX10	.0011	1.409
Ubiquitin fusion degradation protein Ufd1	A0A100I306	.002	1.528
GPI‐anchored cell wall organization protein Ecm33	A0A117DX26	.00025	3.546
l‐asparaginase	A0A117E3D0	.019	2.865
Phosphoesterase	A0A100IRD2	.013	1.365
Translation initiation factor	A0A100INM7	.0004	1.348
Heat shock protein Hsp98/Hsp104/ClpA	A0A100I757	.019	1.363
Calcium channel subunit Cch1	A0A100II07	.0017	1.377
V‐type proton ATPase subunit F	A0A117DX62	.00082	1.465
Catalase	A0A100I1U7	.00056	2.021
NADH‐cytochrome b5 reductase	A0A124BUN3	.0024	1.476
Peroxidase	A0A117DXD4	.00026	2.016
DNA mismatch repair protein Msh2	A0A117E0Y6	.0069	1.423
Mannitol‐1‐phosphate 5‐dehydrogenase	A0A100I4E3	.00049	1.383
Ras‐like protein	A0A117DUH2	.00018	2.781
Cell division control protein 2 kinase	A0A117E082	.0057	1.341
Allergen	A0A124BYU7	.0046	1.497
DNA replication factor C subunit Rfc5	A0A100I4N1	.00069	1.932
Protein GCY	A0A100IL95	.00108	1.365
Heat shock protein SSC1, mitochondrial	A0A124BXS3	.011	1.320
Import inner membrane translocase subunit tim10	A0A100I2H5	.047	1.470
Mitochondrial import receptor subunit	A0A100IIJ7	.00167	1.548
Mitochondrial import inner membrane translocase subunit tim‐16	A0A100IT22	.0033	1.358
Mitochondrial import receptor subunit tom‐40	A0A117DZK5	.015	1.412
Hsp70 chaperone	A0A100IDD7	.0019	1.381
60S acidic ribosomal protein P1	A0A100INH0	.0065	1.829
Profilin	A0A117E1G0	.0116	1.653
Catalase	A0A117DVZ9	.00025	2.547
Allergen Asp F3	A0A124BYH9	.0154	1.485
FK506‐binding protein 2	A0A100I4T5	.016	1.433
Disulfide isomerase	A0A100I4B6	.0045	1.796
Eukaryotic translation initiation factor 5	A0A117E234	.00019	1.326
Prohibitin	A0A100IIG1	.00027	2.080
Zn(II)2Cys6 transcription factor	A0A124BWU7	.0087	1.593
1,3‐beta‐glucanosyltransferase	A0A100INU2	.0042	1.512
C6 zinc finger domain protein	A0A100IM80	.035	1.589
C6 transcription factor	A0A100IQP1	7.49E−05	1.367
Folylpolyglutamate synthase	A0A100IA90	.00042	1.393
Endoplasmic reticulum DnaJ domain protein Erj5	A0A100INL2	.033	1.559
Cyclophilin‐like peptidyl‐prolyl cis‐trans isomerase	A0A100IS83	.0037	1.529
Cell division control protein 42	A0A100IRP8	.012	1.652
60S ribosomal protein L30	A0A100IPI2	.0198	1.584
E3 ubiquitin‐protein ligase	A0A100IHK9	8.96E−05	1.351
Aldehyde dehydrogenase	A0A100IJL1	.0092	1.428
C6 transcription factor	A0A100ISD3	.00015	2.045
Prohibitin	A0A100IPF9	.0027	1.711
Protein disulfide isomerase	A0A100IRZ3	.0044	1.763
Guanine nucleotide‐binding protein subunit alpha	A0A100IIH6	.000062	1.592

Note

Protein names and accession numbers are based on the Uniprot database. The ratio is referring to the relative quantitative results for the T and C groups.

In addition, some of these DEPs were previously reported to play important roles in stress response mechanisms or metabolic processes of fungi, such as glycosylphosphatidylinositol (GPI)‐anchored cell wall organization protein Ecm33 (ratio 3.546), alpha, alpha‐trehalose glucohydrolase TreA/Ath1 (ratio 2.419), 60S acidic ribosomal protein P1 (ratio 1.829), DNA replication factor C subunit 5 (ratio 1.932), disulfide isomerase (ratio 1.796), C6 zinc finger domain protein (ratio 1.589), and cell division control protein 42 (ratio 1.652). In the discussion section, the specific functions of these proteins in fungal cells will be described in detail. Proteins were selected based on their greater quantification ratio (T/C) among the 53 upregulated proteins. In addition, the roles of Hspssc1 (ratio 1.320), Hsp70 chaperone (ratio 1.381), and Hsp98/Hsp104/ClpA (ratio 1.363) in the HSR process, as well as the functions of peroxidase (ratio 2.016) and catalase (ratio 2.02) with regard to ROS scavenging, have been well described in previous studies. Therefore, the function of the Hsp70 chaperone is also described in the discussion section.

### 3.3 KEGG pathway enrichment

The 1,025 DEPs (comparing the T and C groups) were enriched in numerous KEGG pathways. We identified the top 20 KEGG pathway enrichment terms (Figure 4). A more detailed representation of this list of pathways is shown in Table A2. Corrected *p*‐values are listed in the Table S1. The two most significant enrichment pathways were the metabolic (ang01100) and the oxidative phosphorylation (ang00190) pathways. A total of 137 and 15 DEPs were enriched in these two pathways, respectively. Additional significant enrichment pathways identified in the present study were involved in porphyrin and chlorophyll metabolism (ang00860), pyruvate metabolism (ang00620), and the citric acid cycle (TCA cycle) (ang00020).

### 3.4 Label‐free proteomic analysis

We performed a label‐free proteomic analysis to provide additional information to support the iTRAQ labeling results. Based on the result of the label‐free proteomic analysis, we found that 77 proteins (Table A3) were identified in group T (50°C stress) but not identified in group C (30°C control). The reason for this result may be that the protein expression levels of the 77 DEPs in group C were too low for MS detection. Therefore, we compared this result with the results obtained by iTRAQ proteomic analysis. We found that 12 of the 77 DEPs were identified by the iTRAQ proteomic approach, and the relative quantitation ratio (T/C) was obtained for these proteins. The protein expression levels of the 12 DEPs were upregulated; details of the 12 DEPs are as follows: phosphatidylinositol 4‐kinase type II subunit alpha (ratio: 1.497), β‐galactosidase (EC 3.2.1.23) (ratio: 2.131), cell wall protein (fragment) (ratio: 2.338), rhamnogalacturonate lyase A (ratio: 1.807), carboxypeptidase (EC 3.4.16.‐) (ratio: 2.073), similar to An03g03490 (ratio: 1.776), peroxisomal membrane protein Pmp47 (ratio: 1.356), β‐1,6‐glucanase (ratio: 3.898), extracellular α‐glucosidase (ratio: 2.09), similar to An14g02280 (ratio: 1.425), tripeptidyl peptidase A (ratio: 1.699), and integral membrane protein (ratio: 1.457). Because iTRAQ‐based quantitative proteomic analysis has the advantages of sensitivity and reliability, we regarded iTRAQ proteomic analysis as a superior method for the purposes of this study.

**Figure 3:**
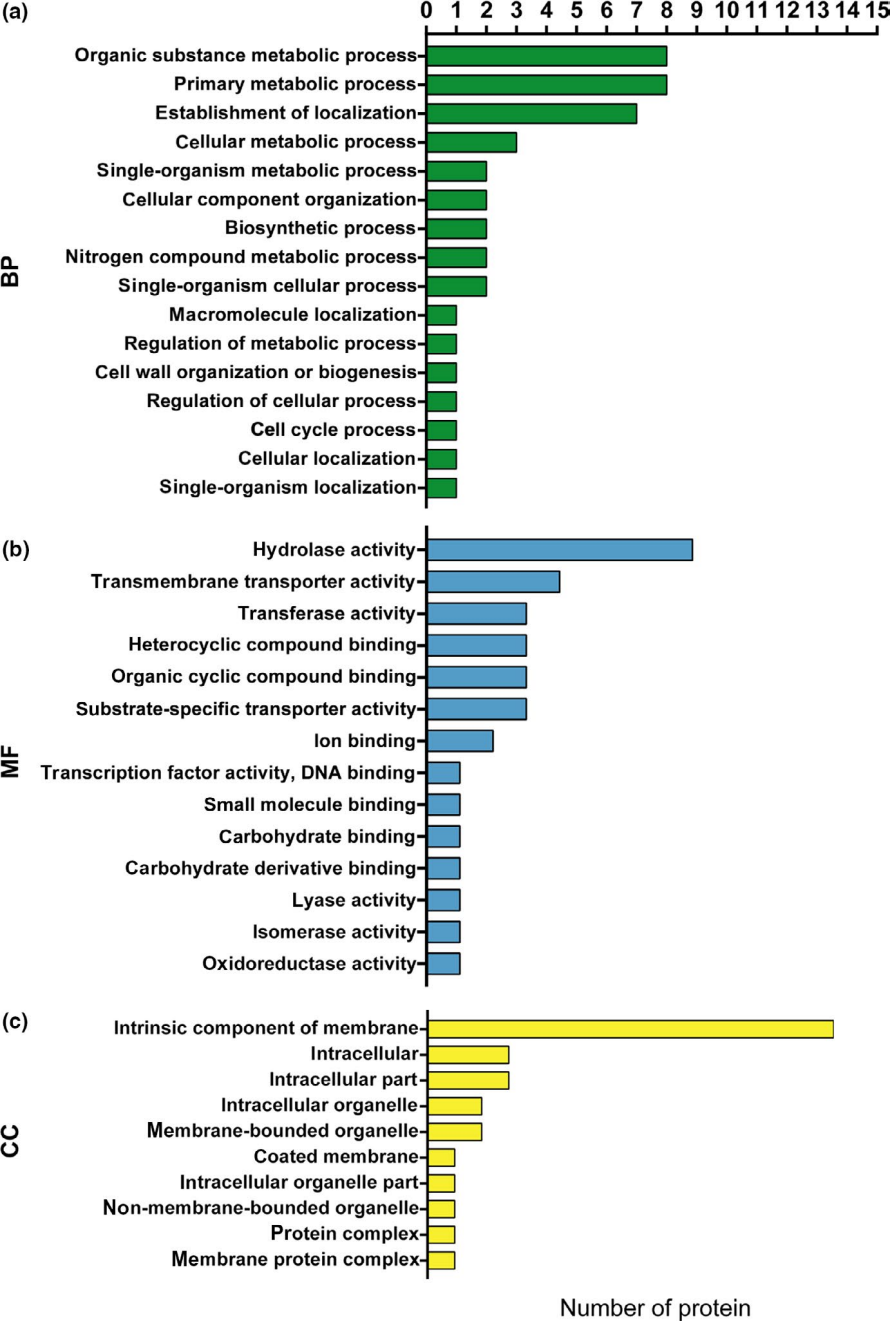
GO enrichment analysis of the 77 differentially expressed proteins (DEPs). GO enrichment terms for DEPs: BP (a), MF (b), and CC (c), representing the biological process, molecular function, and cellular component, respectively. GO enrichment terms (*y*‐axis); number of proteins contained in each GO term (*x*‐axis). (b) The transcription factor activity and DNA binding terms have the same GO ID, and consequently, they are listed in the same row in the figure

We focused on the 77 DEPs because we considered that the protein expression levels of these DEPs had significant differences between groups T and C. Thus, we also performed bioinformatics analysis (GO) on the 77 DEPs. However, the cellular component (CC) and molecular function information for most of the proteins could not be obtained by GO due to a database limitation. Fifteen proteins were identified as intrinsic components of the membrane, based on the main CC enrichment results (Figure 3c).

Fifteen molecular function enrichment terms were identified for 18 proteins (Table 3), and this was further supported by the metadata from the GO database. The following proteins were identified: nine with hydrolase activity, four involved in transmembrane transport, three with transferase activity, three involved in heterocyclic compound binding, three involved in organic cyclic compound binding, three with substrate‐specific transporter activity, two involved in ion binding, and one associated with transcription factor activity, DNA binding, small molecule binding, carbohydrate‐binding, carbohydrate derivative binding, lyase, isomerase, and oxidoreductase activities (Figure 3b).

**Table 3:** List of 18 proteins induced by heat stress and studied by label‐free analysis

Protein name	Accession no.	Alternate ID
Cytochrome P450 61	A0A100I288	ABL_00141
Endopolygalacturonases	A0A100I4L3	ABL_00795
Phosphatidylinositol 4‐kinase type II subunit alpha	A0A100I5B9	ABL_00995
Beta‐galactosidase (EC 3.2.1.23)	A0A100I7G1	ABL_01506
DUF803 domain membrane protein	A0A100I8A3	ABL_01356
Alpha‐1,6‐mannosyltransferase subunit	A0A100IB80	ABL_02446
Peptidyl‐tRNA hydrolase 2	A0A100IBB0	ABL_02482
Sugar transporter	A0A100ID32	ABL_02908
Alpha‐1,6‐mannosyltransferase subunit	A0A100IDU9	ABL_03125
C6 transcription factor	A0A100IKC9	ABL_05017
Nucleoside diphosphatase	A0A100IKH7	ABL_05575
Rhamnogalacturonate lyase A	A0A100IKQ4	ABL_05670
Carboxypeptidase (EC 3.4.16.‐)	A0A100ILU3	ABL_06225
Secreted lipase	A0A100IN59	ABL_06681
Uncharacterized protein	A0A100IP18	ABL_07404
Extracellular serine carboxypeptidase	A0A100IPA5	ABL_07541
Glycosyl hydrolase family 43 protein	A0A100IQ58	ABL_07746
1‐(5‐phosphoribosyl)‐5‐[(5‐phosphoribosylamino)methylideneamino] imidazole‐4‐carboxamide isomerase (EC 5.3.1.16) (5‐proFAR isomerase) (Phosphoribosylformimino‐5‐aminoimidazole carboxamide ribotide isomerase)	A0A100IQ93	ABL_08050

Note

Protein names, accession numbers, and alternate ID are from the Uniprot database. The alternate ID column represents the ID of the protein‐coding genes.

### 3.5 Verification by parallel reaction monitoring

**Figure A1:**
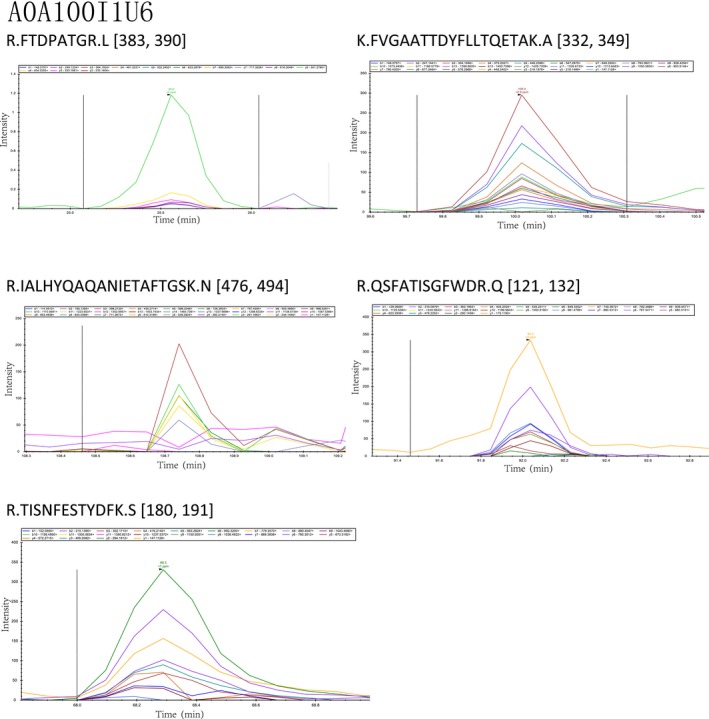
Retention time (*x*‐axis) versus intensity (1E3, *y*‐axis) of each fragment ion corresponding to the candidate peptides of the four selected proteins. The lines with different colors represent different fragment ions. Note: Alpha, alpha‐trehalose glucohydrolase TreA/Ath1 (A0A100I1U6)

**Figure A2:**
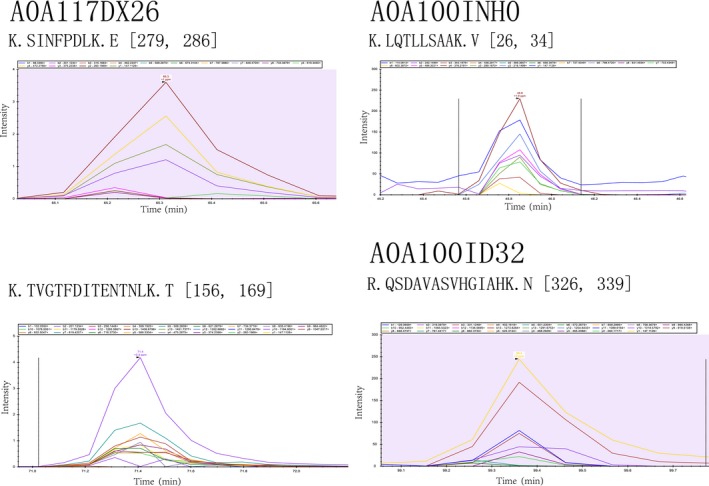
Retention time (*x*‐axis) versus intensity (1E3, *y*‐axis) of each fragment ion corresponding to the candidate peptides of the four selected proteins. The lines with different colors represent different fragment ions. Note: GPI‐anchored cell wall organization protein Ecm33 (A0A117DX26); 60S acidic ribosomal protein P1 (A0A100INH0); Sugar transporter (A0A100ID32)

**Figure A3:**
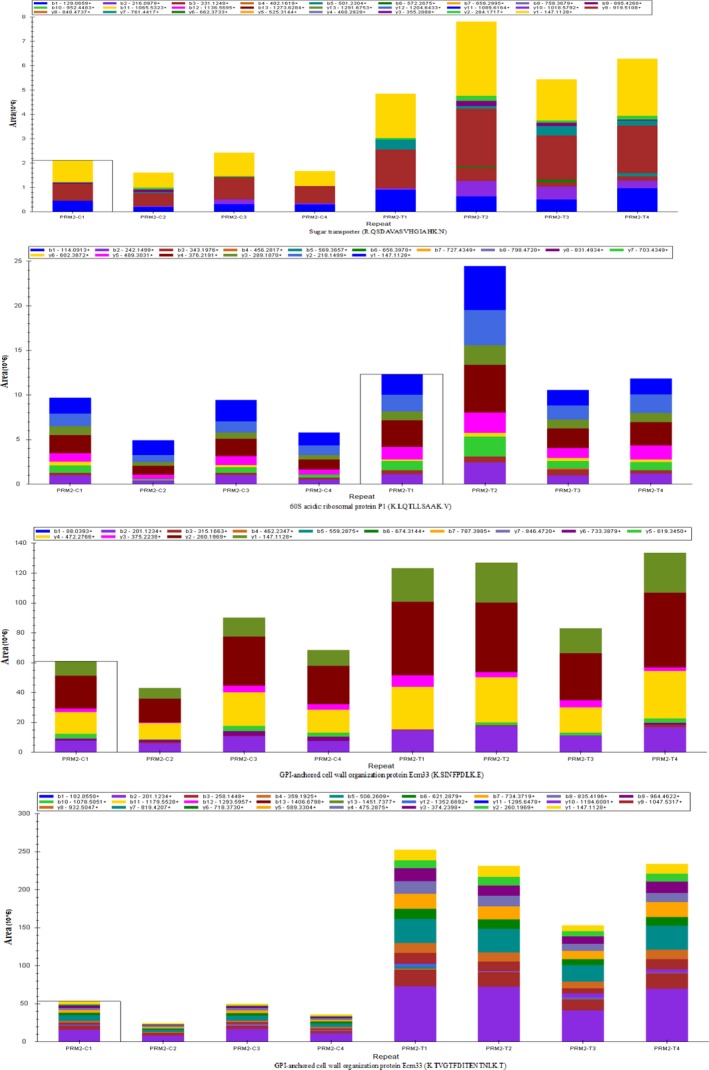
Repeat samples (*x*‐axis) versus area (1E6, *y*‐axis) of each fragment ion corresponding to the candidate peptides of the four selected proteins. The different colors represent secondary fragments (b and y ions). The peptide area is defined as the sum of the areas of all b and y ions of the same peptides

**Figure A4:**
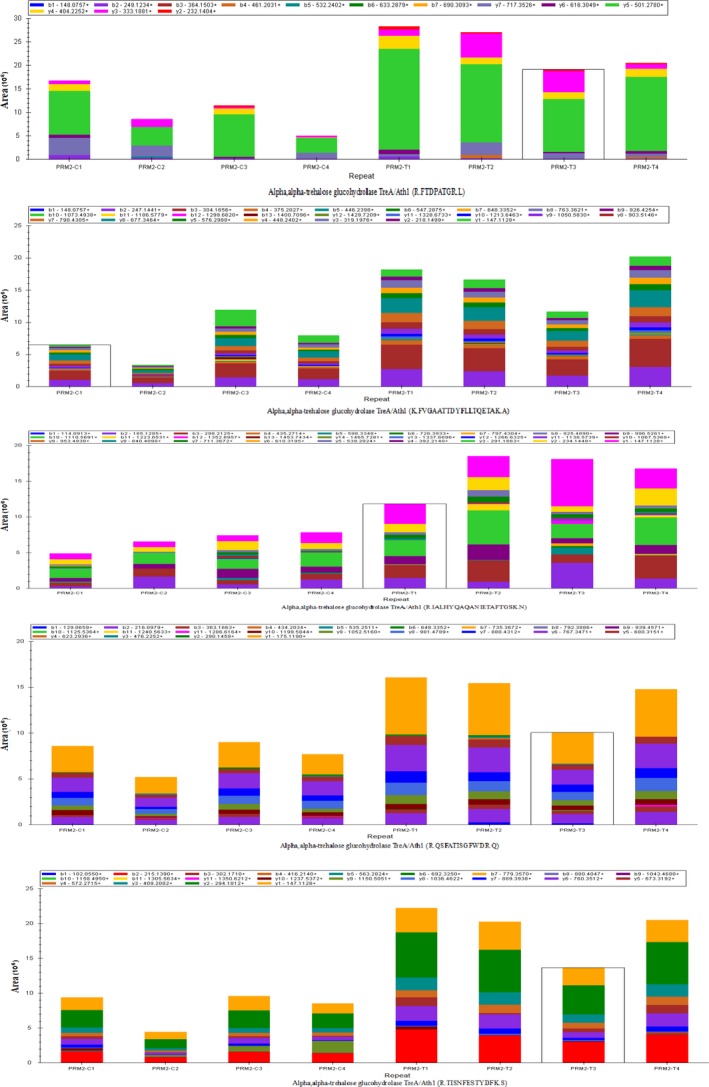
Repeat samples (*x*‐axis) versus area (1E6, *y*‐axis) of each fragment ion corresponding to the candidate peptides of the four selected proteins. The different colors represent secondary fragments (b and y ions). The peptide area is defined as the sum of the areas of all b and y ions of the same peptides

The peptide abundance values of the nine peptides belonging to the selected four proteins were obtained. Analysis results using the Skyline software are shown in Figures A3 and A4. The retention time and intensity of each fragment ion corresponding to the candidate peptides of the four selected proteins are shown in Figures A1 and A2. The results of the PRM analysis indicated that the relative protein expression levels of the selected four proteins were upregulated (Table 4). Compared with the results of proteomic analysis (iTRAQ and label‐free), the relative protein expression levels of the four proteins had the same trends. Therefore, the PRM data further validated the reliability of the proteomic analysis results.

**Table 4:** PRM quantitative results for the four selected proteins

Protein name	Ratio (T/C)	Peptide	*p*‐Value
Source: iTRAQ proteomic analysis			
Alpha, alpha‐trehalose glucohydrolase TreA/Ath1	2.2	R.FTDPATGR.L	.0056
		K.FVGAATTDYFLLTQETAK.A	.0112
		R.IALHYQAQANIETAFTGSK.N	.0098
		R.QSFATISGFWDR.Q	.0071
		R.TISNFESTYDFK.S	.0025
GPI‐anchored cell wall organization protein Ecm33	3.5	K.SINFPDLK.E	.0145
		K.TVGTFDITENTNLK.T	.0003
60S acidic ribosomal protein P1	1.6	K.LQTLLSAAK.V	.0782
Source: label‐free proteomic analysis			
Sugar transporter	3.1	R.QSDAVASVHGIAHK.N	.0008

Note

Ratio (T/C) indicates the peak area ratio of the candidate peptides of each protein.

## 4 DISCUSSION

### 4.1 Functional enrichment analyses

The mechanism of HSR in *A. niger*has not yet been fully described. Previous studies have successfully demonstrated the important role of HSPs and ROS‐scavenging proteins under heat stress at 45°C in *A. niger* (Abrashev et al., 2014). However, the HSR mechanism is complex, and additional proteins are involved in the response process. The heat stress temperature of 50°C used in the present study was higher than that of the temperature used in previous studies. In addition to HSPs and ROS‐scavenging proteins, we provided a comprehensive bioinformatic data analysis on the proteins associated with HSR in *A. niger *3.316. The results of the bioinformatic analyses are useful for exploring the underlying molecular function of DEPs.

Depending on the protein‐specific ratio, the proteins identified in the present study may be closely related to the HSR process in *A. niger *3.316. The molecular functions of these proteins in other fungal cells have been reported. Thus, we concluded that they have the same or similar function in *A. niger *3.316*. *The most highly upregulated protein of the GO terms is GPI‐anchored cell wall organization protein Ecm33, which is localized on the cell surface. The cell walls of the fungi are comprised of three major polysaccharides, namely chitins, glucans, and mannoproteins (Klis, Mol, Hellingwerf, & Brul, 2002; Molina, Gil, Pla, & Arroyo, 2000). Approximately 40% (dry weight) of the cell wall is composed of mannoproteins that serve as a filling material embedded in the chitin and glucan structural network (Pardo et al., 2004). GPI‐anchored cell wall organization protein Ecm33 is vital for appropriate fungal cell wall ultrastructure organization and correct assembly of the mannoprotein outer layer (Pardo et al., 2004). The deletion of the gene encoding this protein can result in a weakened cell wall (Pardo et al., 2004). The main role of alpha, alpha‐trehalose glucohydrolase TreA/Ath1 is to catalyze trehalose hydrolysis; trehalose has previously been identified in *A. niger* (Svanstrom, Leeuwen, Dijksterhuis, & Melin, 2014). The function of trehalose is mainly the prevention of oxidative damage to cells (Pereira, Panek, & Eleutherio, 2003; Singer & Lindquist, 1998). Trehalose is a fungal carbohydrate, and trehalose oxidation has been shown to provide energy for the cells (Novodvorska et al., 2016). Moreover, 60S acidic ribosomal protein P1 is part of the 60S subunit of the fungal ribosome (Wahl & Moller, 2002). A previous study in *Saccharomyces cerevisiae* demonstrated that the deletion of this ribosomal protein affected the activity of yeast ribosomes and altered the selectivity of ribosomes to specific mRNAs (Remacha et al., 1995). Replication factor C subunit 5 (Rfc5) acts as a DNA replication factor in fungi (Sugimoto et al., 1996). Deletion of Rfc5 induces mitosis in yeast cells at certain temperatures, with unevenly separated and fragmented chromosomes (Sugimoto et al., 1996). Based on previous reports, disulfide isomerase is important for protein folding in the endoplasmic reticulum, where it catalyzes the formation and breakage of disulfide bonds in proteins (Wilkinson & Gilbert, 2004). Disulfide bonds stabilize proteins, and their formation is considered the rate‐limiting step in the majority of protein folding processes (Darby, Morin, Talbo, & Creighton, 1995). Moreover, previous studies have demonstrated that the C6 zinc finger domain protein significantly influences the regulation of genes involved in the production of secondary metabolites and the utilization of carbon and nitrogen as substrates (Chang & Ehrlich, 2013). The protein levels of cell division control protein 42 (Cdc42) are regulated by exocytosis and endocytosis (Estravis, Rincon, Portales, & Perez, 2017). This protein is a guanosine triphosphatase (GTPase) involved in the polarization of fungal cells (Estravis et al., 2017). A major consequence of heat stress is the increase in the levels of HSPs. For example, the Hsp70 chaperone protein interacts with partially folded proteins and extended peptide segments to optimize the folding process, regulate protein activity, and prevent aggregation (Mashaghi et al., 2016).

In summary, the information derived from the GO functional enrichment analysis helped us to infer the underlying molecular function of proteins in *A. niger* 3.316 and to aid the understanding of the mechanism of the HSR process.

### 4.2 KEGG pathway analyses

**Figure 4:**
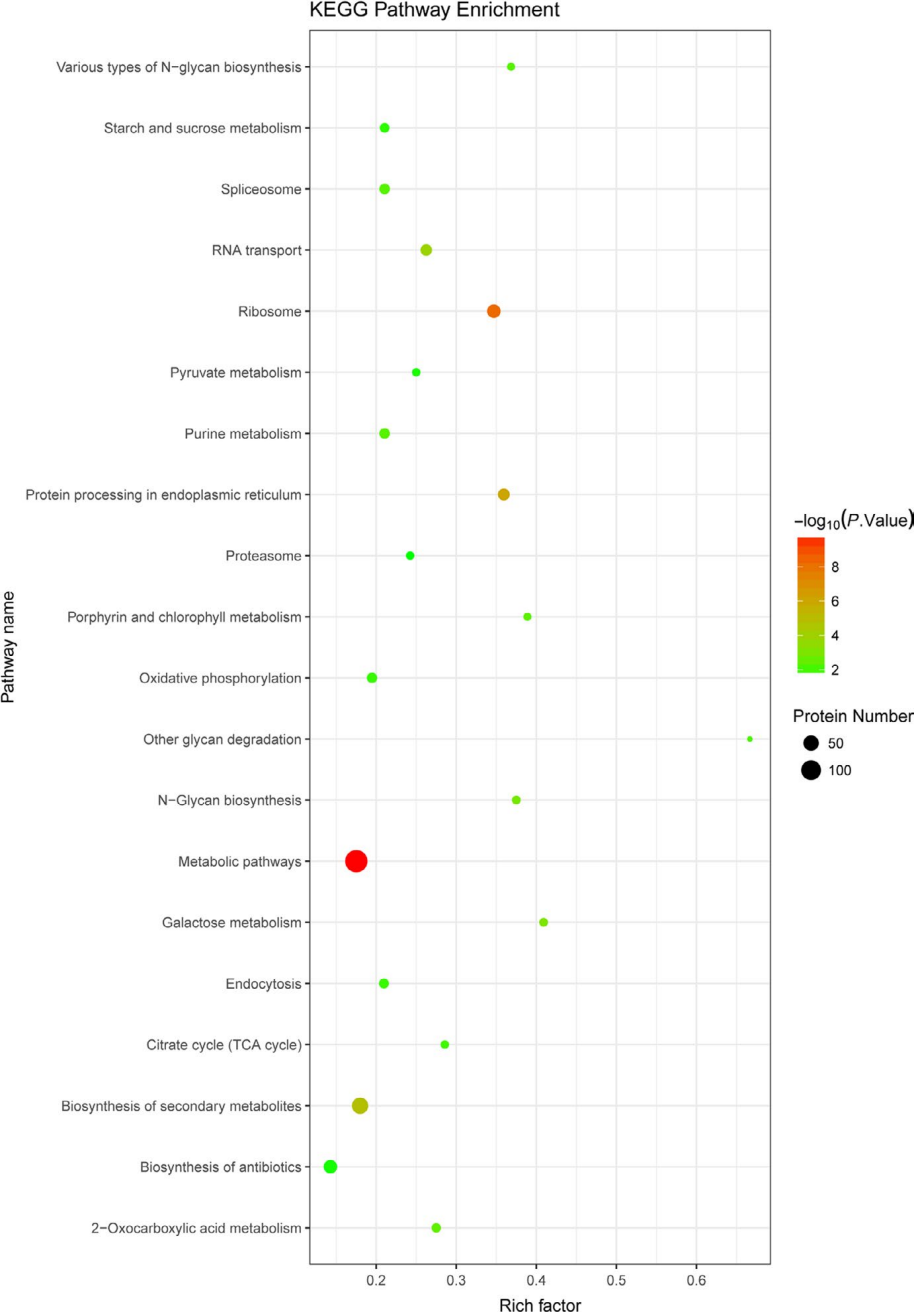
KEGG pathway enrichment of the 1,025 differentially expressed proteins (DEPs). The top 20 pathway enrichment terms (*p* < .05) are displayed; pathway name (*y*‐axis); rich factor (*x*‐axis); each dot represents a KEGG pathway. The color of the dot represents a *p*‐value, and the size of the dot indicates the number of DEPs enriched in the pathway. The rich factor is the ratio of the number of DEPs in one pathway to the number of all background proteins in the same pathway. The larger the rich factor, the more significant the enrichment level of the DEPs in this pathway

The results of the KEGG pathway enrichment analysis can be used to further explore HSR in *A. niger *3.316*. *Most DEPs (Figure 4) were significantly enriched in metabolic pathways, which may illustrate the obvious distinction between the T and the C groups. In addition, the data demonstrated that metabolites in *A. niger *3.316 cells were dramatically altered under heat stress. Furthermore, it is well known that fungi can alter their respiratory pathways during evolution to adapt to new environmental conditions, resulting in different levels of oxidative phosphorylation (Marcet‐Houben, Marceddu, & Gabaldon, 2009). The TCA cycle is a crucial component of respiratory metabolism in both mammalian and fungal cells (Cavalcanti et al., 2014). Thus, under heat stress conditions, oxidative phosphorylation and the TCA cycle increase to maintain cellular energy homeostasis in *A. niger *3.316. In addition, iron (III) porphyrin is involved in the synthesis of catalase (Garcia, Lee, Blasko, & Bruice, 1991). Porphyrin and chlorophyll metabolism directly affect the scavenging of heat‐induced ROS. Moreover, pyruvate is essential for the fungal defense against heat stress and is involved inROS scavenging (Zhang et al., 2017). Therefore, pyruvate metabolism is an important part of the HSR mechanism of *A. niger *3.316.

The KEGG pathway enrichment data provide a guide for further evaluation of protein interactions and functions. However, additional investigation is required regarding the association between HSR and changes in *A. niger *3.316 metabolites.

### 4.3 Bioinformatic analysis of label‐free proteomic data

The GO enrichment results for the 77 DEPs provided an improved understanding of the proteome differences between groups T and C. Although only a small fraction of the 77 DEPs was identified by GO enrichment analysis, the available molecular function information (Table 3) could be used to further understand the HSR mechanism of *A. niger *3.316*. *Fifteen DEPs were identified as intrinsic components of the membrane by GO. This is the main difference between group T and C samples identified by GO. The 15 proteins may be involved in heat stress signal transduction in *A. niger *3.316*. *Furthermore, GO analysis and the results of the iTRAQ proteomic analysis can be combined to more fully describe the differences between groups T and C.

In conclusion, an iTRAQ proteomic approach was applied to compare the proteome changes between group T samples (50°C stress) and group C samples (30°C control), and the observations were further verified using a PRM approach. The results are useful for the understanding of the proteome differences between the two sets of samples. In addition, the label‐free proteomic analysis provided an additional explanation of the differences between groups T and C. Bioinformatic analysis, combined with the proteomics data, significantly enhances our understanding of the HSR mechanism of *A. niger. *In particular, the bioinformatic analysis suggested distinct metabolic patterns between group T samples and group C samples under heat stress conditions, which can aid subsequent metabolomics studies. Further metabolomics studies are needed to investigate detailed metabolite changes to confirm the HSR mechanism of *A. niger*.

## CONFLICT OF INTEREST

None declared.

## AUTHOR CONTRIBUTION

Xiangyu Deng: conceptualization, data curation, formal analysis, investigation, methodology, project administration, resources, software, supervision, validation, writing‐original draft, writing‐review and editing. Bin Du: conceptualization, investigation, project administration, resources, supervision. Fengmei Zhu: conceptualization, investigation, project administration, resources, supervision. Yanan Gao: investigation, resources, software, supervision, validation. Jun Li: conceptualization, funding acquisition, investigation, project administration, resources, supervision. 

## ETHICS STATEMENT

None required.

## Data Availability

The dataset generated and analyzed during the current study is available in the Zenodo repository at http://doi.org/10.5281/zenodo.3608375 (Table S1: KEGG pathway enrichment).

## APPENDIX 1

**Table A1:** Identification of downregulated proteins in the eight molecular function annotations or in the five biological process enrichment terms of GO

Protein name	Accession no.	*p*‐Value	Ratio
60S ribosomal protein L6	A0A100II13	1.78E−05	0.562
Cytidylyltransferase	A0A100IA25	.0019	0.725
Cdc48‐dependent protein degradation adaptor protein	A0A100IMV1	.0006	0.482
tRNA(M5U54) methyltransferase	A0A124BY30	.00084	0.708
cAMP‐dependent protein kinase regulatory subunit	A0A117E2A2	.0038	0.759
Eukaryotic translation initiation factor 3 subunit A	A0A117DUM5	.00065	0.668
S‐(hydroxymethyl) glutathione dehydrogenase	A0A100IUU9	.0199	0.745
Serine/threonine‐protein phosphatase	A0A117DY47	.00058	0.756
Casein kinase II subunit beta	A0A100I9Q1	.0067	0.562
Alpha, alpha‐trehalose‐phosphate synthase	A0A100IQ14	.00019	0.684
Serine proteinase	A0A100IE06	.0122	0.766
Heat shock protein	A0A100IN38	.00029	0.683
Regulator of G protein signaling domain protein	A0A124BY58	.025	0.724
Proteasome maturation factor Ump1	A0A100IKA7	.0076	0.633
Cell division cycle protein 48	A0A124BW77	.00036	0.704
YagE family protein	A0A100I816	.0378	0.643
Nucleoside diphosphate kinase	A0A100IMC3	.005	0.758
Rho GDP‐dissociation inhibitor	A0A100INZ0	.000308	0.569
Coproporphyrinogen III oxidase	A0A100ICH3	.0047	0.584
Serine palmitoyltransferase 2	A0A124BY35	.0013	0.641
Actin‐related protein 2/3 complex subunit 1A	A0A117E328	.00296	0.747
Snf1 kinase complex beta‐subunit Gal83	A0A117DZG7	.00041	0.710
Glutaredoxin	A0A100IHH4	.0022	0.753
Flap endonuclease 1	A0A100IPI4	7.36E−05	0.589
RNA annealing protein Yra1	A0A124BXK7	.000317	0.399
Genome maintenance protein MGM101	A0A117E2B3	.0065	0.739
Translocation protein	A0A100ILT5	.0034	0.643
Presequence translocase‐associated motor subunit Pam17	A0A100I369	.0214	0.711
Prefoldin subunit 6	A0A100IKZ0	.0067	0.719
Tubulin‐specific chaperone Rbl2	A0A124BZ06	2.51E−05	0.640
Nascent polypeptide‐associated complex subunit alpha	A0A124BX09	2.31E−05	0.613
60S acidic ribosomal protein P2	A0A100IL88	.00028	0.543
Regulatory protein SUAPRGA1	A0A100IKZ5	.00049	0.722
Flocculation suppression protein	A0A100IKV5	.0087	0.747
RNA polymerase II Elongator subunit	A0A100IH59	.00094	0.474
40S ribosomal protein S2	A0A100ISE5	.0027	0.603
Retrograde regulation protein 2	A0A100IIV8	.036	0.577
C6 finger domain protein	A0A124BV29	.0356	0.752
40S ribosomal protein S28	A0A117E3C1	.0007	0.518
Transcription elongation factor S‐II	A0A117E2R0	.0048	0.668
Hsp90 co‐chaperone Cdc37	A0A100IBI0	4.71E−05	0.735

Note

The protein name, alternate ID and accession number were from the Uniprot database. The ratio of the column indicated the ratio of the protein quantitative results in the T and C groups.

**Table A2:** KEGG pathway analysis data of *A. niger *3.316 under heat stress

Pathway name	ID	Number of DEPs	Background number	*p* Value
Metabolic pathways	ang01100	137	782	1.16E−10
Ribosome	ang03010	34	98	5.52E−09
Protein processing in endoplasmic reticulum	ang04141	23	64	9.86E−07
Biosynthesis of secondary metabolites	ang01110	59	328	1.55E−05
RNA transport	ang03013	21	80	.00014
Galactose metabolism	ang00052	9	22	.00099
*N*‐Glycan biosynthesis	ang00510	9	24	.00162
2‐Oxocarboxylic acid metabolism	ang01210	11	40	.00395
Porphyrin and chlorophyll metabolism	ang00860	7	18	.00451
Various types of *N*‐glycan biosynthesis	ang00513	7	19	.00572
Purine metabolism	ang00230	16	76	.00576
Spliceosome	ang03040	16	76	.00576
Other glycan degradation	ang00511	4	6	.00794
Citrate cycle (TCA cycle)	ang00020	8	28	.01101
Endocytosis	ang04144	13	62	.01247
Oxidative phosphorylation	ang00190	15	77	.01323
Starch and sucrose metabolism	ang00500	12	57	.01561
Pyruvate metabolism	ang00620	8	32	.02054
Biosynthesis of antibiotics	ang01130	34	238	.02121
Proteasome	ang03050	8	33	.02363

Note

The number of DEPs column indicates the number of differentially expressed proteins enriched in this KEGG pathway. The background number column indicates the total number of proteins involved in this KEGG pathway under heat stress conditions. The ID from the KEGG database.

Abbreviation: TCA, tricarboxylic acidcycle.

**Table A3:** The induction of protein expression by heat stress

Protein name	Accession no.	Alternate ID	MF result
Cytochrome P450 61	A0A100I288	ABL_00141	Yes
WD domain protein	A0A100I2Y8	ABL_00329	
Similar to An01g06420	A0A100I370	ABL_00399	
Caffeine‐induced death protein Cid2	A0A100I3D3	ABL_00452	
Endopolygalacturonases	A0A100I4L3	ABL_00795	Yes
Uncharacterized protein	A0A100I4U1	ABL_00859	
Phosphatidylinositol 4‐kinase type II subunit alpha	A0A100I5B9	ABL_00995	Yes
Beta‐galactosidase (EC 3.2.1.23)	A0A100I7G1	ABL_01506	Yes
DUF803 domain membrane protein	A0A100I8A3	ABL_01356	Yes
Uncharacterized protein	A0A100I8J8	ABL_01790	
Cell wall protein (Fragment)	A0A100I9B8	ABL_02002	
Centromere/kinetochore protein Zw10	A0A100IA46	ABL_02155	
PAP2 domain protein	A0A100IAM1	ABL_02302	
Alpha‐1,6‐mannosyltransferase subunit	A0A100IB80	ABL_02446	Yes
Peptidyl‐tRNA hydrolase 2	A0A100IBB0	ABL_02482	Yes
Sugar transporter	A0A100ID32	ABL_02908	Yes
Nuclear protein Qri2/Nse4	A0A100IDU0	ABL_03131	
Alpha‐1,6‐mannosyltransferase subunit	A0A100IDU9	ABL_03125	Yes
Integral membrane protein	A0A100IEK7	ABL_03129	
Uncharacterized protein	A0A100IJJ7	ABL_05078	
C6 transcription factor	A0A100IKC9	ABL_05017	Yes
Nucleoside diphosphatase	A0A100IKH7	ABL_05575	Yes
Rhamnogalacturonate lyase A	A0A100IKQ4	ABL_05670	Yes
Carboxypeptidase (EC 3.4.16.‐)	A0A100ILU3	ABL_06225	Yes
Similar to An03g03490	A0A100IMQ8	ABL_06732	
Peroxisomal membrane protein Pmp47	A0A100IN48	ABL_06577	
Secreted lipase	A0A100IN59	ABL_06681	Yes
Mannosylphosphorylation protein	A0A100IN87	ABL_06986	
Transcription factor SipA3	A0A100IN99	ABL_07015	
Uncharacterized protein	A0A100IND5	ABL_07044	
OPT oligopeptide transporter	A0A100INQ3	ABL_07205	
Similar to An11g09480	A0A100INV3	ABL_07314	
Uncharacterized protein	A0A100IP18	ABL_07404	Yes
Heat shock Hsp30‐like protein	A0A100IP71	ABL_07177	
Similar to An12g06000	A0A100IPA0	ABL_07538	
Extracelular serine carboxypeptidase	A0A100IPA5	ABL_07541	Yes
Glycosyl hydrolase family 43 protein	A0A100IQ58	ABL_07746	Yes
1‐(5‐phosphoribosyl)‐5‐[(5‐phosphoribosylamino) methylideneamino] imidazole‐4‐carboxamide isomerase (EC 5.3.1.16) (5‐proFAR isomerase) (Phosphoribosylformimino‐5‐aminoimidazole carboxamide ribotide isomerase)	A0A100IQ93	ABL_08050	Yes
Short chain dehydrogenase	A0A100IR96	ABL_08376	
RING finger ubiquitin ligase	A0A100IRE2	ABL_08603	
Similar to An13g01520	A0A100IT82	ABL_09548	
Uncharacterized protein	A0A100ITC5	ABL_09626	
Carboxypeptidase Y	A0A100ITF4	ABL_09655	
Transmembrane 9 superfamily member	A0A100IU63	ABL_10030	
Uncharacterized protein	A0A100IUC6	ABL_10167	
Alpha‐amylase	A0A100IUD4	ABL_10224	
GPI‐anchor biosynthesis protein	A0A100IUI8	ABL_10265	
Amine oxidase (EC 1.4.3.‐)	A0A100IUL5	ABL_10344	
ATP synthase subunit alpha	A0A100IUM3	ABL_10325	
Uncharacterized protein	A0A117DUN0	ABL_00268	
Isoamyl alcohol oxidase	A0A117DVP7	ABL_01121	
Beta‐1,6‐glucanase	A0A117DVW2	ABL_01196	
PHD finger domain protein	A0A117DWI9	ABL_01711	
Proteasome subunit beta type (EC 3.4.25.1)	A0A117DX69	ABL_02137	
Extracellular alpha‐glucosidase	A0A117DZ97	ABL_02512	
Signal peptide peptidase	A0A117DZD7	ABL_03839	
MBOAT family protein	A0A117DZK1	ABL_04003	
Similar to An14g02280	A0A117E0S6	ABL_05581	
Copper‐transporting ATPase	A0A117E0X3	ABL_05808	
Extracellular cellulase CelA/allergen Asp F7‐like	A0A117E1I3	ABL_06779	
Allantoin permease	A0A117E1L5	ABL_06889	
Xyloglucan‐specific endo‐beta‐1,4‐glucanase A	A0A117E1L6	ABL_06886	
Tripeptidyl peptidase A	A0A117E271	ABL_07771	
alpha‐1,2‐Mannosidase (EC 3.2.1.‐)	A0A117E298	ABL_07876	
Integral membrane protein	A0A117E3L3	ABL_09106	
Amidohydrolase 2	A0A117E3M4	ABL_10004	
Similar to An12g10400	A0A117E4P7	ABL_10097	
Uncharacterized protein	A0A124BUQ6	ABL_00297	
Mitochondrial carrier protein	A0A124BV04	ABL_00775	
Phosphatidate cytidylyltransferase (EC 2.7.7.41)	A0A124BWC1	ABL_02796	
Similar to An15g07560	A0A124BWS8	ABL_03489	
Cell wall protein PhiA	A0A124BXN4	ABL_05613	
SNARE domain protein	A0A124BXX7	ABL_06434	
Similar to An03g04840	A0A124BY20	ABL_06829	
Carboxylic ester hydrolase (EC 3.1.1.‐)	A0A124BYN7	ABL_08774	
Carboxylic ester hydrolase (EC 3.1.1.‐)	A0A124BYX3	ABL_09572	
Phospholipase C PLC‐C	A0A124BYX9	ABL_09584	

Note

The protein name, accession number and alternate ID were from the Uniprot database. The MF column indicated the proteins that acquired molecular function information. The alternate ID column represents the ID of the protein‐codinggenes.
